# Translating Treg Therapy in Humanized Mice

**DOI:** 10.3389/fimmu.2015.00623

**Published:** 2015-12-14

**Authors:** Susanne A. Hahn, Iris Bellinghausen, Bettina Trinschek, Christian Becker

**Affiliations:** ^1^Department of Dermatology, University Medical Center, Johannes Gutenberg-University, Mainz, Germany

**Keywords:** humanized mice, Treg function, therapy, autoimmune disease risk, tolerance

## Abstract

Regulatory T cells (Treg) control immune cell function as well as non-immunological processes. Their far-reaching regulatory activities suggest their functional manipulation as a means to sustainably and causally intervene with the course of diseases. Preclinical tools and strategies are however needed to further test and develop interventional strategies outside the human body. “Humanized” mouse models consisting of mice engrafted with human immune cells and tissues provide new tools to analyze human Treg ontogeny, immunobiology, and therapy. Here, we summarize the current state of humanized mouse models as a means to study human Treg function at the molecular level and to design strategies to harness these cells for therapeutic purposes.

## The Emergency of Treg as Therapeutic Target

The notion that T cells can be actively involved in immunological tolerance goes back to the seventies of the last century when it was shown that T cells (thymus-derived lymphocytes) are required for tolerance induction and could transfer tolerance to naïve recipients (1, 2). Tolerance induction by T cells has thereafter been convincingly demonstrated by numerous investigators using a variety of experimental systems (3, 4). Despite decades of research, it was not possible to unequivocally identify the responsible T cell population. In the 90s, Simon Sakaguchi and colleagues were able to prove that a small population of constitutively CD25-expressing CD4^+^ T cells prevents autoimmune disease in mice (5). The discovery of the X chromosome encoded transcription factor Foxp3 (forkhead box protein 3) (6–9) as a specification and maintenance factor (10, 11) subsequently confirmed CD4^+^CD25^+^ T cells as a unique thymus-derived lineage. A population of phenotypically and functionally similar T cells could also be corroborated in humans (12, 13). Examining their role in the murine immune system revealed that regulatory T cells (Treg) – as they had been called in foresight – take a central role in immune homeostasis: genetic defects that turn Treg dysfunctional result in multiorgan autoimmune disease (14) and their depletion induces autoimmunity (15). The investigation of their suppressive activity was greatly helped by major improvements in molecular profiling techniques and revealed a number of potential suppressive mechanisms (16). More recently, several Treg effector molecules were found to play a role in Treg peripheral expansion and activation rather than suppressive activity (17) or contribute to Treg function only under particular conditions (18). Moreover, only few mechanisms have been confirmed in mice and man (19–21).

While Treg were initially investigated mainly in regard to their effects on T cells, their far-reaching regulatory activity affects the activation, differentiation, and survival of all types of immune cells. Although their main role appears to be the prevention of autoimmune reactions their broad immune-regulatory potential and bystander activation in immune responses affects a wide range of conditions such as infection, cancer, transplantation, allergy, and inflammatory diseases, but also responses toward foreign antigens, as well as fetal antigens during pregnancy (22–28).

Murine and human Treg, like all T cells, consist of numerous differentiated subpopulations (29–38). While in some cases, the functional properties of subpopulations have been described in detail, a systematic assignment of phenotypes and functions is missing in both species. Strikingly, beyond their role in immune homeostasis and immune cell regulation, specialized Treg subpopulations also interfere with non-immunological processes such as metabolism (39) and wound healing (36, 40, 41). So far, the latter populations have so far exclusively been described in mice.

To date, the vast majority of data concerning Treg were obtained from mouse experiments. The reason for this is the fact that human tissue cannot always be investigated. However, human Treg studies are also technically limited by the lack of a reliable and specific Treg marker molecule (21, 42). Whereas in healthy subjects, the main human Treg population in blood can at least be approximated as CD45RO^+^TCR^+^CD4^+^CD25^high^Foxp3^+^CD127^neg^ cells (43), their analysis and isolation remains affected by similarly phenotyped non-regulatory T helper cells under conditions of T cell activation such as in inflammatory disease (44, 45). Today, the most effective, reliable, and objective criteria for the identification and quantification of Treg in diagnostic or therapeutic settings is in quantitative real-time PCR-based methylation analysis of an evolutionarily conserved element within the Foxp3 locus (46, 47). This method however only indirectly contributes to Treg isolation from human samples due to associated cell destruction.

Their potent and far-reaching regulatory activities have helped Treg to become a major subject in virtually all fields of medical research. With regard to the lack of a comprehensive understanding of human Treg composition and function, a cautionary approach to Treg modulation in patients appears to be warranted, even more so, as animal models failed to predict effects of Treg modulating agents in humans (48). The latter, in particular, has made clear that human Treg function as well as Treg targeted immune intervention need to be examined more closely. As these studies cannot be performed in man, a model organism is required in which the function of human cells can be examined outside the human body. In recent years, humanized mouse models have emerged as a suitable tool to study human Treg function and to develop strategies for their therapeutic application.

## Treg in Autoimmune Disease and Cancer

The main physiological role of Treg appears to be the prevention of autoimmune responses (49) as evidenced by fulminate autoimmune reactions in their absence (50) (Table 1). In turn, their autoprotective activity suggests that autoimmune diseases may result from their functional impairment. While their peripheral number remains unchanged in the majority of autoimmune diseases (51–53), their suppressive function seems to be indeed altered (51, 52, 54–56). However, retrospective studies in patients are confused with the problem that Treg dysfunctionality can be either intrinsic or reflect changes in other immune components (57–61). The autoimmune phenotype of scurfy mice, for example, results from both Treg dysfunction and an increased population of self-reactive T cells emerging from progenitor cells which did not properly develop into Treg. Thus, to reveal the functional state of Treg and to understand their role and possible effects in disease, causes and effects need to be carefully discerned. As shown by Trinschek et al. (62), humanized mouse models can help to elucidate disease mechanisms by enabling to individually manipulate and combine diseased and unaffected Treg from patients and healthy donors. It needs to be mentioned that in addition to the thymus-derived Treg population non-regulatory CD4^+^ T cells can acquire a similar regulatory function and phenotype outside of the thymus, typically under the influence of particular forms of inflammation (63). Here, we focus on thymus-derived Treg since they are the most widely studied in humanized mice.

**Table 1:** **Reported Treg alterations in autoimmune diseases**.

Disease	Source	Observations	Reference
Multiple sclerosis	Blood	Normal frequencies, decreased suppressive function	(53, 64–66)
	Cerebral spinal fluid	Enhanced frequency, memory phenotype	
Rheumatoid arthritis	Blood	Contradictory results on frequencies	(67, 68)
		CD62^+^Treg appear functionally impaired	
	Inflamed joint	Enhanced frequencies	
Type I diabetes	Blood	Contradictory results on frequencies	(69, 70)
Crohn’s disease	Blood	Contradictory results on frequencies	(71, 72)
		Enhanced Foxp3^+^IL-17^+^ T cells	
Systemic lupus erythematosus	Blood	Reduced frequencies correlate with enhanced disease activity	(51, 73)
Psoriasis	Blood	Contradictory results	(74–76)
	Psoriatic plaques	Enhanced Foxp3 expression	

Regardless of numerous genetic changes, tumors are primarily the body’s own tissue (77). Their destruction by the immune system therefore represents – at least in part – an autoimmune response. Since Treg appear to be in part self-reactive (78), it is not surprising that increased Treg activity is causally involved in tumor-induced tolerance (52, 79). Numerous studies reported elevated numbers of Treg in the tumor tissue, lymphoid tissues, and peripheral blood of cancer patients (43, 52, 79–81), also the infiltrating of Treg into the tumor site correlates with poor prognoses in many tumor types (82). Some mechanisms of tumor-associated Treg recruitment and activation have been proposed (83–85), but may vary between tumor types and/or stages. Whereas Treg depletion increases all inflammatory responses including antitumor immunity (86), it is effective only in early stages of tumor growth (87, 88). Despite its proven genuine potential as a strategy to overcome tumor tolerance, Treg depletion is not yet a therapeutic option in cancer patients. While animal experiments strongly indicate that only a complete and selective Treg destruction may affect antitumor immunity, Treg depletion in cancer patients is hampered by inefficient non-specific depleting agents, which cause unwanted side effects (89). On the other hand, current treatment options such as immune checkpoint blockade seem to affect the Treg compartment (90). Thus, further investigation on the interaction of Treg and tumor cells in humans are needed and reagents for effective and selective Treg destruction need to be identified.

## Humanized Mouse Models: Challenges and Limitations to Study Human Treg Biology

**Figure 1:**
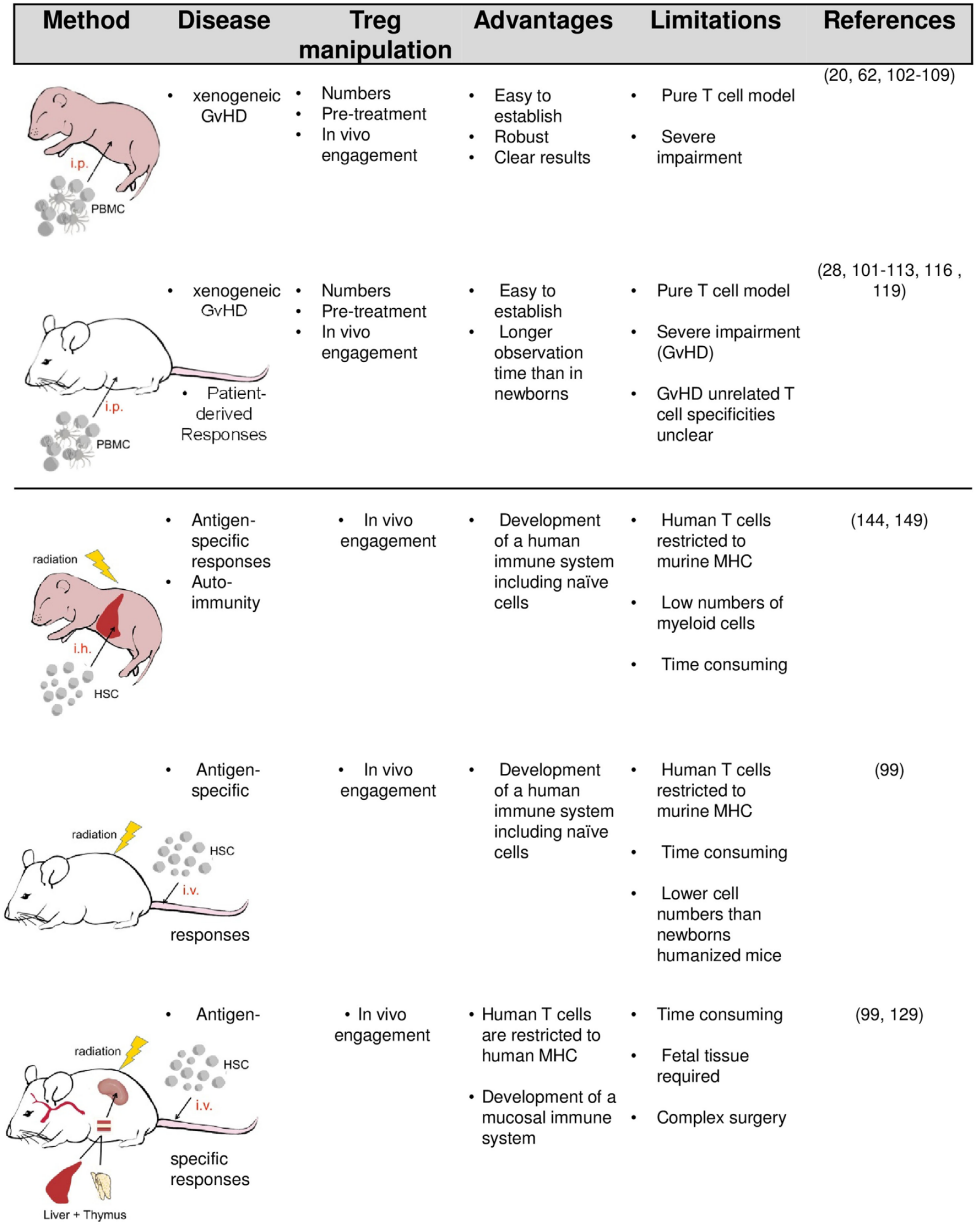
**Humanized mouse models and their potential application in Treg research**.

The simplest way to equip mice with human immune cells (mainly PBMC) is their intravenous or intraperitoneal injection (Figure 1). In order to accept the human graft, the recipients need to be immunodeficient and the degree of deficiency strongly influences the survival and function of transplanted human cells. Initial experiments by McCune and Mosier in severe combined immunodeficient (*SCID*) mice lacking T and B cells revealed that human immune cells can survive in mice for several weeks (91, 92). However, due to the remaining innate immunity and leakiness in adaptive cell development in aged SCID mice, transplanted human cells are only transiently accepted. Reduced natural killer cell (NK cell) activity in non-obese diabetic (NOD)-*SCID* mice improve engraftment. Profound and lasting impairment of adaptive and innate immunity by targeted mutation of the IL-2R gamma-chain gene in NOD-SCID IL-2Rγ^−/−^ (NSG) or BALB/c recombinase activating gene (Rag)2^−/−^ IL-2Rγ^−/−^ mice eventually enable a stable long-term survival of transplanted human cells and tissues (93–99).

Upon engraftment of human PBMC into immunodeficient mice, a population of human T cells reacts against murine MHC molecules, supposedly on murine antigen-presenting cells, resulting in T cell-driven lethal xenogeneic graft-versus-host disease (GvHD) (92, 94, 100). In contrast to T cells, other transferred immune compartments such as myeloid cells, dendritic cells, or B cells rapidly vanish or at least become irretrievable. In adult NOD-*SCID*, sublethal irradiation or depletion of innate murine immune cells (called “conditioning”) accelerates GvHD onset. In contrast, GvHD can be reliably induced in newborn NOD-*SCID* and gamma-chain-deficient recipients without conditioning.

Graft-versus-host disease upon transfer of human PBMC is characterized by weight loss (or lack of weight gain in pubs), reduced mobility and a total mortality >95% (20). At the tissue level, it is accompanied by a massive infiltration of human T cells into all mouse organs leading to peribronchial and perivascular inflammation and increased mucus production, colitis, skin rashes, and increased glutamate pyruvate transaminase (GPT) serum levels indicative of hepatitis. When performed in newborn NOD-*SCID* mice, GvHD leads to death within 30–90 days, depending on the number of transferred PBMCs. Interestingly, the number of transferred T cells and their functional state influences disease onset: both increased cell numbers and cells from autoimmune patients accelerate disease onset (62). While increased cell numbers contain more xenoreactive cells, the reason for the latter effect is not completely clear. Supposedly, disease-mediated alterations in the T cell compartment, cross-reactivity or increased inflammatory cytokine production may act beneficial.

**Figure 2:**
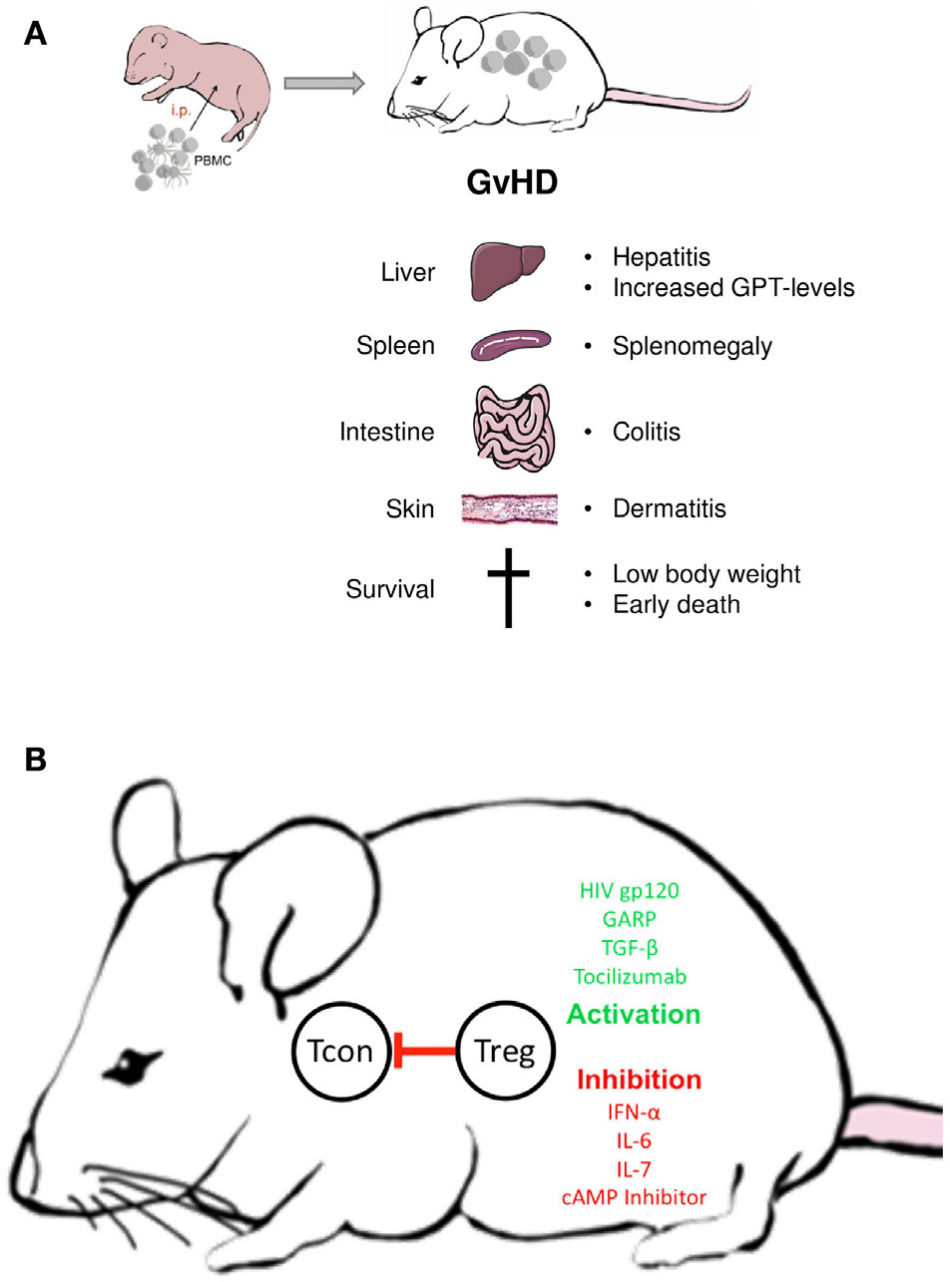
**Treg manipulation in the xenogeneic GvHD model**. **(A)** Disease symptoms; **(B)** examples of biologicals with demonstrated Treg activating or inactivating activity in the GvHD model; Tcon = conventional non-regulatory T cell.

Owing to its simplicity, the xenogeneic GvHD provides a robust means to functionally evaluate Treg outside the human body (Figure 2): Without further treatment, the limited number of Treg transferred within the PBMC do not affect GvHD onset. However, transfer of additional Treg in ratios between 4:1 and 10:1 (PBMC:Tregs) suppresses all GvHD symptoms in a dose-dependent manner (20, 101). Since the suppressive activity of Treg is not antigen specific, either syngenic or allogeneic Treg may be used. Treg ratios lower than 10:1 appear ineffective, however, allowing to test biologicals with modest Treg-activating potential (102).

Differences exist between mouse strains in regard to the persistence of Treg-mediated GvHD suppression. While in NOD-*SCID* mice, Treg-induced tolerance remains stable for weeks (20), it is only transient in NOD-*SCID*γc^−/−^ or *Rag*2γc^−/−^ mice (102, 103). Thus, while all three strains can be used to investigate human Treg activation *in vivo*, a long-term evaluation of the functional state is only possible in the later model. Not only Treg numbers but also certain biologicals have been shown to selectively increase or repress Treg function in the transfer model (20, 104–106). Particularly, strong stimuli can prevent the xenogeneic response without increasing the number of Treg in PBMC (20). Alternatively, Treg can be separately pretreated with modulating agents or pathway inhibitors before transfer (107, 108). The latter approach is especially helpful in testing compounds which lack Treg specificity. Similar to *in vitro* assays, the GvHD model can be used to discern patient-associated functional alterations in Treg or T effector cells by combining either cells from patients or healthy donors (62).

Without Treg activation, the xenogeneic GvHD cannot be prevented. It is however delayed by the transfer of very low cell numbers and greatly reduced in MHC class I or class II knockout NOD-*SCID*γc^−/−^ or *Rag*2γc^−/−^ mice (100, 109, 110). In this manner and by the use of PBMC from patients’ inflammatory responses such as allergy-induced inflammation and its regulation by Treg can be studied (102, 111–114). In the same manner, the transfer model has been used to analyze the mechanism of wound healing, tissue remodeling, and allograft rejection after tissue transplantation. Several investigators reported that the transfer of Treg prevented the rejection of human skin, islets, bronchus, and arterial grafts (96, 115–119). In addition to the general effect of Treg on the disease, these models can be used to compare differently isolated or expanded Treg populations (28, 120). So far, almost only total CD4^+^CD25^+^ populations have been used, followed by approval of disease repression, but without looking at their further development and survival.

Since current animal models may not accurately identify cancer immunotherapies with clinical potential, reliable preclinical tools are needed to test these drugs directly against human cancers. Here again, in its simplest form, a humanized mouse tumor model can be set up by cotransplantation of human immune cells and tumors. The first difficulty in setting up such a model consist in the constitutive dependency of primary human tumor cells on host growth factors, which greatly limits their ability to grow in a different species. Alternatively, long-term established tumor cell lines engraft well and are readily available. Depending on their origin and methodology of cultivation human tumor cell lines can phenocopy primary tumors (121, 122). Their growth in immunodeficient mice therefore provides a robust cell line model system that potentially predicts patient responses to drugs or biologicals (123, 124).

Adding human immune cells either by transplantation of immune cells or stem cells, does however, complicates the setting. Cotransplantation of human immune cells and allogeneic tumor cell lines creates the need to distinguish between xenogeneic, allogeneic, and potential tumor-specific responses. Although tumor-specific responses may be selectively followed by MHC complex multimers, they will be cross-influenced by strong inflammatory xenogeneic and/or allogeneic background activities. Therefore, a more promising approach consists in a syngeneic approach. In a syngeneic setting, CD8^+^ CTL have been shown to effectively combat patient tumor cells in immunodeficient mice (125, 126), and such models may help to identify tumor rejection antigens and T cell receptors for redirecting immunity to tumors in a patient-individualized manner. Although more complicated to set up, they may also provide a basis to study the interaction between patient-derived Treg and tumor cells and to develop reagents for effective and selective Treg impairment.

Another way to humanize mice is to transfer hematopoietic stem cells (HSC) either alone or in combination with human tissues required in lymphocyte development (Figure 1). Upon transfer of HSC into newborn or adult mice, multiple lineages of human hematopoietic cells (including T and B cells, plasmacytoid cells, myeloid cells, and NK cells) develop, resulting in a mixed murine/human CD45^+^ cells compartment. Even greater levels of human cell engraftment are achieved when human fetal tissue is cotransplanted. In the BLT (bone marrow/liver/thymus) model, human fetal liver and autologous thymus fragments are implanted under the renal capsule and HSC cells isolated from the same fetal liver are separately transferred to engraft the bone marrow.

Whereas in HSC-humanized mice, human T cells populate the murine thymus and become H2 restricted, they appear to remain HLA restricted in the BLT model (99, 127–129). To achieve HLA-restriction in the former, multiple HLA-transgenic NSG mice have recently been developed (110, 128, 130–133). HSC-based humanized mouse models have also been further modified by replacing distinct murine genes with the corresponding human alleles using knock-in technology, or the transcription activator like effector nuclease (TALEN) technology (134, 135). Each human factor improves the development of certain human hematopoietic lineages. Administration of human IL-15, for example, promotes human NK cell development, while human IL-7 enhances the maintenance of immature thymocytes (136–138). Similarly, transient expression of human GM-CSF and IL-4, macrophage colony-stimulating factor, or erythropoietin and IL-3 (SGM3) increase the reconstitution of DC, monocytes/macrophages, and erythrocytes, respectively (135, 136, 139). In membrane-bound human stem cell factor (SCF)-expressing NSG mice, the differentiation of immature and mature granulocytes including c-Kit^+^ human mast cells has been described (140).

In stem cell-transplanted humanized mice, the developing human T cell compartment includes CD4^+^CD25^+^Foxp3^+^ Treg. Interestingly, the transfer of human CD34^+^ HSC into NSG-SGM3 mice-expressing transgenes for human SCF, GM-CSF, and IL-3 not only led to elevated human myeloid cell frequencies in blood, spleen, bone marrow, and liver compared with non-transgenic NSG recipients, but also to a significant increase in the CD4^+^CD25^+^Foxp3^+^ Treg population. Furthermore, the authors demonstrated Treg expansion in the periphery and not in the thymus as the numbers of CD4^+^Foxp3^+^ thymocytes were similar in both mouse strains. Human DC subsets capable of regulating Treg cell frequencies were suggested as one possible mechanism (141, 142). In these models, Treg have been shown to suppress polyclonally activated T cell proliferation (141) and can be engaged to prevent fibrosis (143).

Unfortunately, like in the transfer models, HSC-humanized mice develop GvHD (144–146). Onset of GvHD is not prevented by the lack of murine MHC molecules and develops despite the presence of human Treg. The development of GvHD in apparently all models limits the experimental window of other immune responses and shows that the possible involvement of GvHD, even if it cannot be detected based on established parameters and its possible influence on other immune responses needs to be taken into account.

Considering the many differences between the species, the growth and survival of human cells in mice is still a surprising find. Many, if not the majority of murine cytokines and growth factors, do not bind or insufficiently interact with the respective human receptors, and the cells need to adapt to this situation. In fact, on closer characterization, proliferating xenoresponsive human T cells differentiate into unusual subsets in the xenogeneic environment (147, 148). Moreover, phenotypical and functional analyses of lymphoid lineages in humanized mice >20 weeks posthematopoietic stem cell transplantation reveals that CD8^+^ T cells and NK cells exhibited functional impairments (149). These observations remind that in humanized mouse models, the mere presence of certain cell types cannot be taken for their functional property. It is, for example, not known whether the cellular and molecular mechanisms that govern the differentiation of the Treg population in mice also applies to HSC-based models (150). While in the mouse, the composition of the Treg population is significantly influenced by commensals, nothing is known in the humanized mouse models (151). Thus, in order to translate potential Treg therapy into the clinic, the origin, fate, and function of Treg in humanized mice must be examined in greater detail.

In summary, in their current state, transfer models provide a robust means to evaluate the interaction of Treg with T cells, particularly in conjunction with Treg activating or inactivating reagents. HSC-based humanized mouse models offer greater possibilities but are difficult to set up and less well explored with regard to Treg development and function. Observations in SGM3 mice are encouraging, and further outfitting mice with additional human factors may help to overcome existing limitations, which might lead to models in where human Treg function and development can be studied in a physiological manner.

## Author Contributions

SH and BT: substantial contributions to the conception and design of the work; IB and CB: substantial contributions to the conception of the work.

## Conflict of Interest Statement

The authors declare that the research was conducted in the absence of any commercial or financial relationships that could be constructed as a potential conflict of interest.
